# Pharmacological or genetic inhibition of LTCC promotes cardiomyocyte proliferation through inhibition of calcineurin activity

**DOI:** 10.21203/rs.3.rs-3552794/v1

**Published:** 2023-11-30

**Authors:** Lynn A.C. Devilée, Jessica M. Miller, Janice D. Reid, Abou Bakr M. Salama, Qinghui Ou, Madiha Jamal, Yibing Nong, Douglas Andres, Jonathan Satin, Tamer M. A. Mohamed, James E. Hudson, Riham R. E. Abouleisa

**Affiliations:** 1 QIMR Berghofer Medical Research Institute, Cardiac Bioengineering Laboratory, Brisbane, Queensland, Australia; 2 School of Biomedical Sciences, Faculty of Health, Queensland University of Technology, Brisbane, Queensland, Australia; 3 Institute of Molecular Cardiology, Department of Medicine, University of Louisville, KY, U.S.A.; 4 Surgery Department, Baylor College of Medicine, Houston, TX, U.S.A; 5 Faculty of Medicine, Zagazig University, Zagazig, Egypt; 6 College of Medicine, Alfaisal University, Riyadh, Saudi Arabia; 7 Center for Cardiometabolic Science, Christina Lee Brown Envirome Institute, Department of Medicine, University of Louisville, Louisville, KY, U.S.A.; 8 Department of Molecular and Cellular Biochemistry, University of Kentucky College of Medicine, Lexington, KY, U.S.A; 9 Department of Physiology, University of Kentucky College of Medicine, Lexington, KY, U.S.A; 10 School of Biomedical Sciences, The University of Queensland, Brisbane, Queensland, Australia

**Keywords:** LTCC, RRAD, Calcium, Cardiomyocyte proliferation, Cell cycle, calcineurin

## Abstract

Cardiomyocytes (CMs) lost during ischemic cardiac injury cannot be replaced due to their limited proliferative capacity, which leads to progressive heart failure. Calcium (Ca^2+^) is an important signal transducer that regulates key cellular processes, but its role in regulating CM proliferation is incompletely understood. A drug screen targeting proteins involved in CM calcium cycling in human embryonic stem cell-derived cardiac organoids (hCOs) revealed that only the inhibition of L-Type Calcium Channel (LTCC), but not other Ca^2+^ regulatory proteins (SERCA or RYR), induced the CM cell cycle. Furthermore, overexpression of Ras-related associated with Diabetes (RRAD), an endogenous inhibitor of LTCC, induced CM cell cycle activity in vitro, in human cardiac slices, and in vivo. Mechanistically, LTCC inhibition by RRAD induces the cell cycle in CMs by modulating calcineurin activity and translocating Hoxb13 to the CM nucleus. Together, this represents a robust pathway for regenerative strategies.

## Introduction

Fetal cardiomyocytes (CMs) proliferate to achieve organ growth during development, but once matured, CMs typically become post-mitotic, permanently exit the cell cycle and stay in the G0 phase^1^. As a result, the regenerative capacity of the heart is limited, which hinders the treatment of ischemic heart failure. Therefore, identifying the factors that control cell cycle arrest in CMs is crucial to re-initiate their dormant proliferative capacity. Calcium (Ca^2+^) is a critical regulator of CM function. During excitation-contraction coupling, Ca^2+^ enters CMs through the LTCC, triggering Ca^2+^ release from the sarcoplasmic reticulum (SR) through ryanodine receptors (RYR). Then, during relaxation, Ca^2+^ is transported back into the SR by the SR Ca-ATPase (SERCA) and out of the cell by the sodium-calcium exchanger (NCX) and the plasma membrane calcium ATPase pump (PMCA) ^2^. Besides regulating contraction, Ca^2+^ signaling controls other activities, including gene expression and transcription, metabolism, survival, cardiac development, and maturation ^3–7^. Therefore, investigating how cardiac Ca^2+^ handling impacts cell cycle activity will provide new insights into the role of Ca^2+^ signaling in cardiac regeneration. LTCC are initiators of Ca^2+^ cycling in CMs, and a recent study suggested that small molecule inhibition of LTCC activity is a potential target for induction of CM proliferation, although how this activates cell cycle activity is unknown ^8^. The LTCC contains five subunits: the pore-forming subunit, α1, and different auxiliary subunits, α2, β, γ, and δ. The α1 subunit allows the passage of Ca^2+^ ions through LTCC into CMs, while the auxiliary subunits modify the function of the channel ^9^. Ras-related associated with Diabetes (RRAD) is a member of the RGK family of Ras-related small G proteins and an endogenous regulator of LTCC activity. RRAD binds directly to the β subunit of the LTCC, thereby controlling LTCC current (I_Ca, L_) ^10,11^. Exogenous RRAD overexpression potently inhibits I_Ca,L_ in adult and embryonic ventricular myocytes ^11,12^. However, whether RRAD impacts the ability of CMs to proliferate is still unknown. Recent studies have provided insights into the downstream impact of Ca^2+^ signaling, highlighting the role of the calcium-dependent serine/threonine phosphatase calcineurin in mediating cell-cycle arrest of postnatal CMs ^7,13^. Calcineurin is localized in the plasma membrane in the vicinity of Ca^2+^ channels. Calcineurin directly links Ca^2+^ signaling to protein phosphorylation status upon increased intracellular Ca^2+^ and plays an essential role in numerous signaling processes ^14–17^. This includes proliferation, where forced expression of activated calcineurin (CnA) causes a premature switch from hyperplasic to hypertrophic growth. In contrast, genetic ablation of calcineurin B prolongs the window of CM proliferation and induces the cell cycle in mature CMs partly through the induction of nuclear translocation of the transcription factor Hoxb13 ^7,13^. In this study, we describe how pharmacological or RRAD-mediated inhibition of LTCC promotes CM division in vitro and in vivo through inhibition of calcineurin activity.

## Results

### Small molecule screen targeting proteins involved in CM Ca^2+^ cycling identifies nifedipine, an LTCC inhibitor, as an activator of the CM cell cycle.

**Figure 1:**
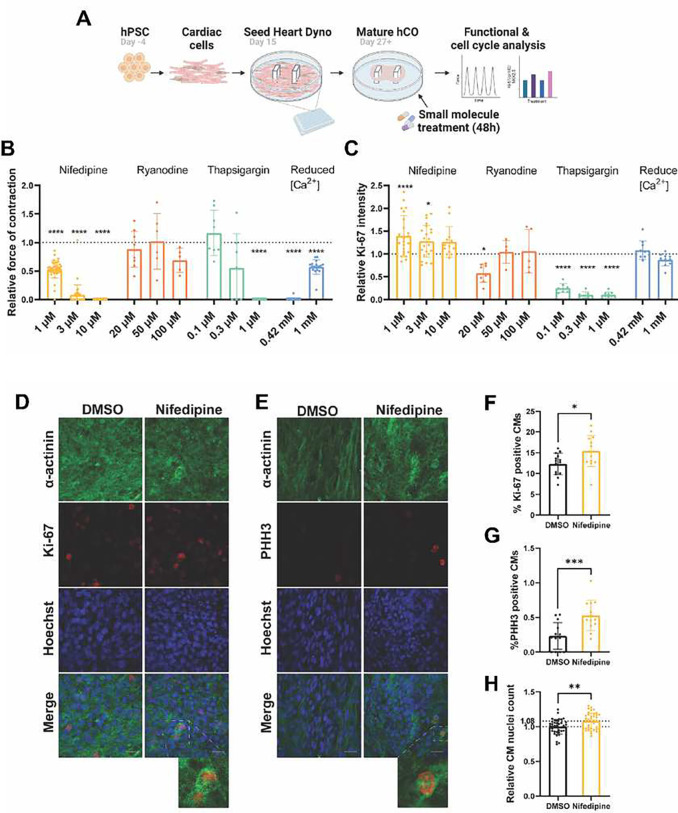
Drug screen targeting proteins involved in CM Ca^2+^ cycling revealed that inhibition of LTCC enhances CM cell cycle activity in hCOs. **(A)** Schematic diagram of hCO formation in a high throughput 96-well plate format and experimental setup. **(B)** Bar graph representing the relative force of contraction in hCOs treated with nifedipine (n= 17–38 hCOs in 2–4 experiments), ryanodine (n= 5–9 hCOs in 1 experiment), thapsigargin (n= 7–9 hCOs in 1 experiment) or a reduced [Ca^2+^] in the medium (n= 17–18 hCOs in 2 experiments) for 48 h. **(C)** Bar graph representing relative Ki-67 intensity across the whole hCO treated for 48 h with nifedipine (n= 13–23 hCOs in 2–4 experiments), ryanodine (n= 5–8 hCOs in 1 experiment), thapsigargin (n= 7–9 hCOs in 1 experiment) or a reduced [Ca^2+^] in the medium (n= 10 hCOs in 2 experiments). **(D)** Representative images of hCOs treated with DMSO (vehicle) or nifedipine for 48 h and stained against α-actinin (green), Hoechst (blue), and Ki-67 (red) or **(E)** PHH3 (red). Bar graphs representing the percentage of CMs positive for **(F)** Ki-67 (n=14 hCOs from 3 experiments), or **(G)** PHH3 (n=13–14 hCOs from 3 experiments) (Scale bar 20 μm). **(H)** Relative CM nuclei count based on NKX2.5 positive immunostaining (n= 38–39 hCOs from 4 experiments). The relative data is normalized to DMSO-treated hCOs. Data are presented as mean ± SD with individual data points representing individual hCOs. *P<0.05, **P<0.01, ***P<0.001, ****P<0.0001.

To assess how disruption of the Ca^2+^ transient impacts CM cell cycle activity, we performed a drug screen targeting proteins involved in CM Ca^2+^ cycling in our cell cycle matured human cardiac organoids (hCOs)^18^. hCOs were treated with nifedipine (LTCC inhibitor), ryanodine (RyR inhibitor), thapsigargin (SERCA inhibitor), or reduced extracellular Ca^2+^ levels ([Ca^2+^]_e_) for 48 h and hCO function and cell cycle activity were assessed (Fig. 1A). Although Reducing Ca^2+^ flow into the CMs using nifedipine or a varying [Ca^2+^]_e_ dose-dependently reduced force of contraction (Fig. 1B), only nifedipine induced cell cycle activity in mature hCOs, as indicated by a significant increase in relative Ki-67 intensity (Fig. 1C). Disrupting sarcoplasmic reticulum Ca^2+^ flux by ryanodine or thapsigargin had a more variable impact on the function and cell cycle activity (Fig. 1B). To validate the effect of nifedipine on inducing cell cycle in CMs, hCOs were treated with either DMSO (vehicle) or 3μM nifedipine for 48 h. Cell cycle induction was assessed by quantification of the percentage of Ki-67 positive CM nuclei (overall cell cycle marker), the percentage of PHH3 positive CM nuclei (G2-M phase marker), and the count of NKX2.5 positive nuclei (CM nuclei marker). Nifedipine treatment significantly increased the percentages of CMs positive for cell cycle markers Ki-67 and PHH3 compared to DMSO-treated hCOs. Moreover, CM nuclei number was significantly increased by ~8% in nifedipine-treated hCOs compared with DMSO-treated hCOs (Fig. 1D–H). We confirmed our findings in primary CMs isolated from P7 mice pups (NMCM P7). NMCM P7 treated with nifedipine showed a significant increase in PHH3 positive CM nuclei and CM nuclei number compared with vehicle-treated CMs **(Supplementary Figure S1)**. These data suggest that inhibition of LTCC using nifedipine promotes CM cell cycle induction in hCOs and primary CMs.

### Single-cell RNA seq in P1 NMCM showed that RRAD, an endogenous inhibitor of LTCC, is highly expressed in spontaneously proliferating cardiomyocytes.

**Figure 2:**
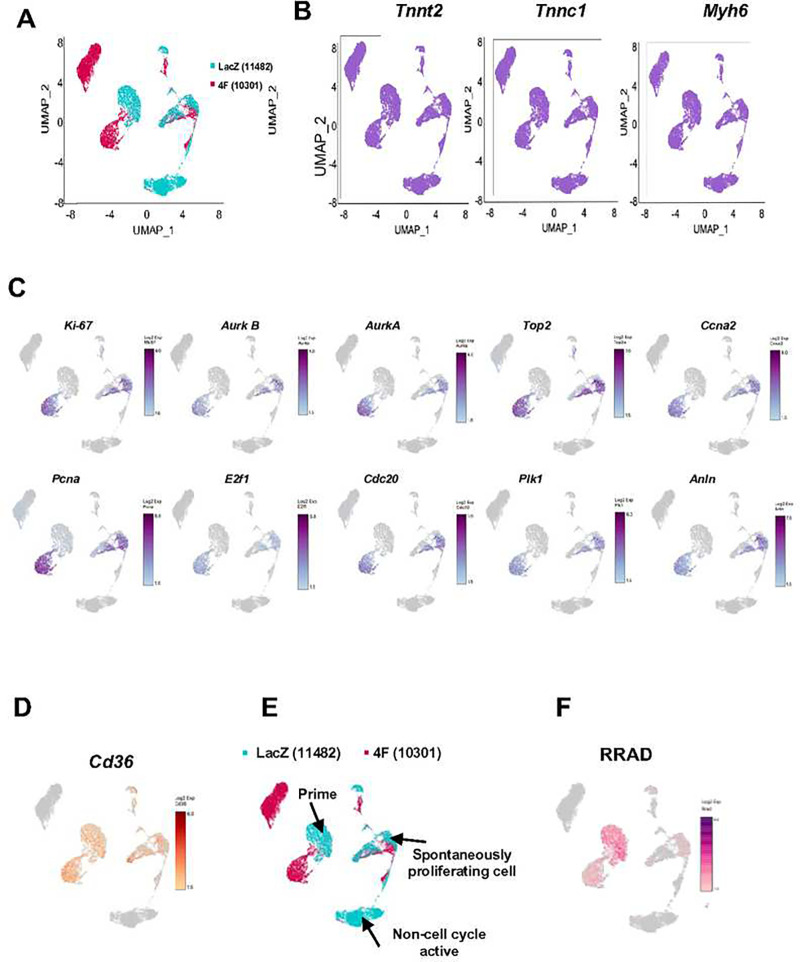
Single Cell RNAseq from P1 NMCM demonstrated RRAD is highly expressed in spontaneously proliferating CMs and CMs that respond to cell cycle induction. **(A)** UMAP plots displaying global gene expression for all single CMs sequenced 24h post LacZ (control) or 4F transduction (n=11482, 10301 respectively). **(B)** All cells show high expression of the cardiac markers *Tnnt2*, *Tnnc1*, and *Myh6*, indicating a pure CM population. **(C)** P1 NMCM that showed high expression of mitotic/cytokinesis genes (*Ki-67*, *Aurora Kinase B* and *A*, *Top2*, *Ccna2*, *Pcna*, *E2f1*, *Cdc20*, *Plk1*, and *Anln*). **(D)** P1 NMCM showed high expression of *Cd36*, a marker for CMs that are more likely to respond to cell cycle stimulation. **(E)** Re-clustering based on the expression of the mitotic genes and the expression of *Cd36* generated 3 clusters - the spontaneously proliferating CMs, primed CMs, and non-cell cycle active CMs **(F)** RRAD expression colocalized within the spontaneous proliferating CMs and the primed CMs.

Due to the limitation of using a small molecule inhibitor, which includes the possibility of off-target effects, in this study, we tried to identify a regulator of LTCC of which the expression correlates with cell cycle activity in CMs. Therefore, we performed single-cell RNAseq of P1 mouse CMs that had not yet exited the cell cycle (naturally proliferating) with and without induction of cell cycle activity using Cdk1/CyclinB1 and Cdk4/CyclinD1 complexes (referred to as “4F”), which was previously shown to lead to cell cycle induction in CMs ^19,20^. Gene expression data were collected from ~11000 cells/condition as summarized in UMAP blots (Fig. 2A). Cardiac specificity was confirmed based on the expression of cardiac markers (*Tnnt2*, *Tnnc1*, and *Myh7*) (Fig. 2B). A unique cell population in the LacZ treated (control) CMs expressed high levels of mitosis/cytokinesis genes (Mki67, *Aurka*, *Aurkb*, *Top2*, *Ccna2*, *Pcna*, *E2f1*, *Cdc20*, *Plk1*, and *Anln*); this population was identified as the spontaneously proliferating CMs (Fig. 2C). Our recent publication revealed *Cd36* as a marker for CMs that can be primed to proliferate ^19^. *Cd36* was highly expressed in the spontaneously proliferating CMs as well as the population of CMs that responded to 4F cell cycle induction (Fig. 2D). Therefore, we divided CMs transduced with LacZ into 3 clusters: spontaneously proliferating CMs where mitotic genes were highly expressed, primed CMs which responded to forced cell cycle induction (4F), and non-cell cycle active CMs that did not express cell cycle genes nor respond to cell cycle stimulation (Fig. 2E). When focusing on genes related to Ca^2+^ signaling, *RRAD*, which is an endogenous specific inhibitor of the LTCC, was identified to be highly expressed in the spontaneously proliferating and primed CM populations (Fig. 2F). These data suggest that *RRAD* expression positively correlates with CM cell cycle activity.

### RRAD overexpression promotes CM division in NMCM P7.

**Figure 3:**
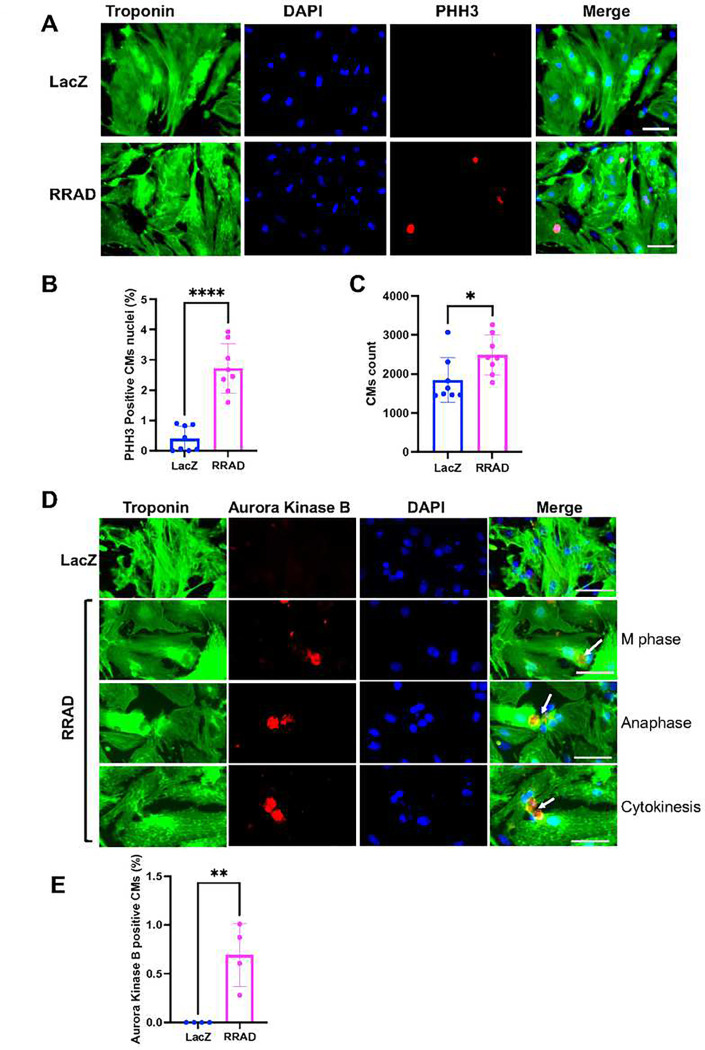
RRAD overexpression in NMCM P7 promotes cell cycle induction. **(A)** Representative images of NMCM P7 transduced with LacZ or RRAD adenovirus and stained against troponin-T (green), PHH3 (red), and Dapi (blue) (Scale bar 100μm). **(B)** Bar graph of CM counts**. (C)** Quantification of the percentage PHH3 positive CM nuclei (n=8), **(D)** Representative images of NMCM P7 transduced with LacZ or RRAD adenovirus and stained against troponin-T (green), Aurora Kinase B (red), and Dapi (blue) (Scale bar 50μm). **(E)** Quantification of the percentage Aurora Kinase B positive CM nuclei (n=4). Data are presented as mean ± SD, *p<0.05, **p<0.01, ****p<0.0001.

To investigate the effect of RRAD overexpression on CM cell cycle induction, first, we transduced NMCM isolated from P7 mouse pups with LacZ (control) or RRAD adenovirus for 72 h. We confirmed RRAD overexpression in P7 NMCM using qRT-PCR and Western blotting **(Supplementary Figure S2)**. RRAD overexpression promoted CMs to enter the cell cycle, as indicated by a significant increase in PHH3-positive CM nuclei compared with LacZ-treated CMs. A significant increase in NMCM P7 CM number was also observed in RRAD-overexpressing CMs compared to LacZ-control CMs (Fig. 3A–C). To confirm that RRAD overexpression promotes complete CM cell division, we assessed the expression of Aurora Kinase B, a cytokinesis marker, using immunostaining. NMCM P7 overexpressing RRAD showed a significant increase in CMs stained positive for Aurora Kinase B compared to the control NMCM (Fig. 3D, E). These results indicate that inhibition of LTCC through RRAD overexpression promotes CM cell division in P7 NMCM.

### RRAD overexpression primes more CMs to proliferate in response to cell cycle stimuli.

Overexpression of CCNA2^21^, a combination of CCND and CDK4 (2F)^20^, or a combination of CDK1/CDK4/CCND/CCNB (4F)^19,20^ have been demonstrated to drive cell cycle induction in CMs. To examine whether RRAD can prime more CMs to respond to such cell cycle stimuli, NMCM P7 were transduced with RRAD or LacZ adenovirus for 24 h and subsequently transduced with adenoviruses encoding different cell cycle stimulators, CCNA2, 2F, or 4F for another 48 h. A significant increase in PHH3 positive nuclei (a marker for the G2-M Phase) and EDU positive nuclei (a marker for the G1-S Phase) compared with CCNA2, 2F, or 4F alone was observed **(Supplementary Figure S3**). These data indicate that inhibition of LTCC by RRAD can successfully prime more CMs to re-enter the cell cycle in response to cell cycle stimuli.

### RRAD overexpression promotes CM cell division and primes more CMs to proliferate in response to cell cycle stimuli in adult human heart slices.

**Figure 4:**
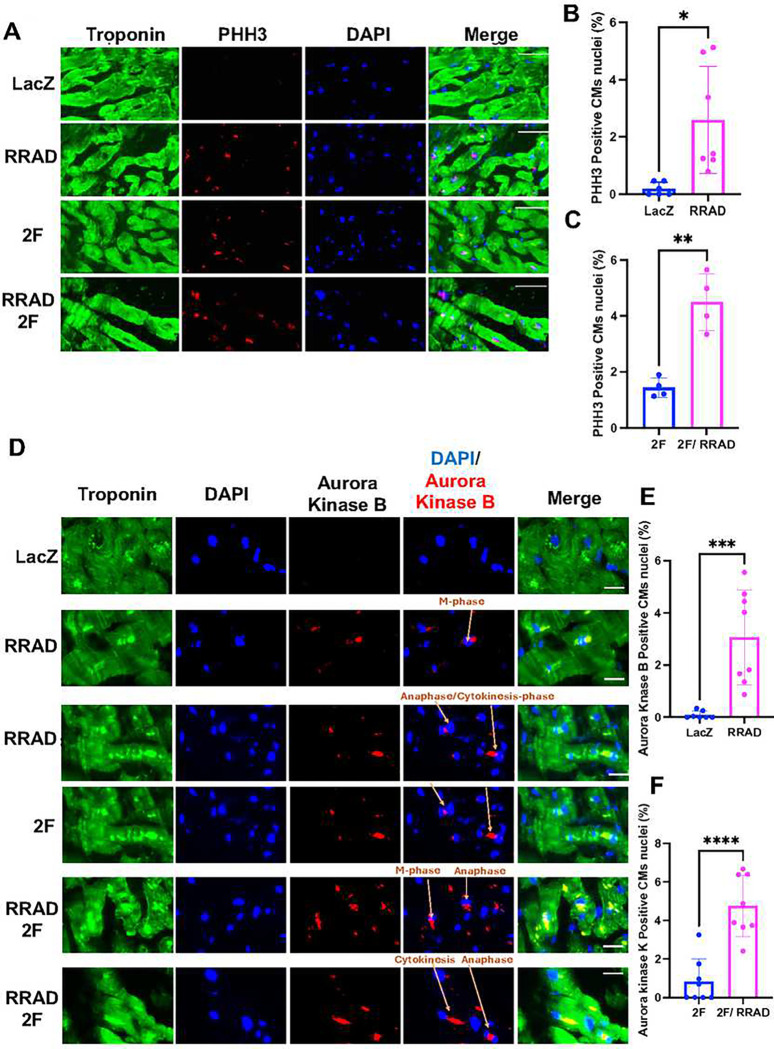
RRAD overexpression in human heart slices promotes cell cycle induction and induces more CMs to enter the cell cycle in response to cell cycle stimuli. **(A)** Representative images of human heart slices transfected with LacZ or RRAD adenovirus for 24 h, then transfected with CDK4 and CCND (2F) for 48 h and stained against troponin-T (green), PHH3 (red) and Dapi (blue) (Scale bar 20μm). **(B-C)** Quantification of the percentage PHH3 positive CM nuclei (n=4–6 individual slices). **(D)** Representative images of human heart slices transduced with LacZ or RRAD adenovirus for 24 h, then transduced with CDK4 and CCND (2F) for 48 h and stained against troponin-T (green), Aurora Kinase B (red), and Dapi (blue) (Scale bar 20μm). **(E-F)** Quantification of the percentage of Aurora Kinase B positive CM nuclei (n=8). Data are presented as mean ± SD, * p<0.05, **p<0.01, ***p<0.001, ****p<0.0001.

To examine the effect of RRAD overexpression on adult primary CMs *ex vivo*, human heart slices ^22^ were infected with adenoviruses encoding LacZ or RRAD for 72 h. RRAD overexpression was confirmed using RT-PCR (**Supplementary Figure 4A)**. 72 h post-infection, RRAD overexpression promoted cell cycle induction in human heart slices as indicated by a significant increase in CM nuclei stained positive for PHH3 versus LacZ-treated heart slices (Fig. 4A–B). To investigate the effect of RRAD overexpression on priming more CMs to enter the cell cycle in response to 2F, human heart slices were infected with LacZ or RRAD adenovirus for 24 h and then infected with 2F adenoviruses for 48 h. RRAD overexpression in human heart slices augmented more CMs to enter the cell cycle, as indicated by a significant increase in PHH3-positive nuclei compared to 2F-treated heart slices (Fig. 4A, C). To validate the mitotic entry of the adult CMs, human heart slices that overexpressed RRAD were stained for Aurora Kinase B. We observed a significant increase in CM nuclei stained positive for Aurora Kinase B compared to LacZ-control heart slices (Fig. 4D, E). Furthermore, human heart slices that overexpressed RRAD in combination with 2F showed a significant increase in CM nuclei stained positive for Aurora Kinase B compared to the heart slices that overexpressed 2F alone (Fig. 4D, F). RRAD overexpression can thus effectively induce and prime mature primary human CMs to re-enter the cell cycle *ex vivo*.

Next, we used the functional readouts of the hCOs to assess how RRAD overexpression affects cell cycle dynamics and functionality of hCOs. hCOs were transduced with LacZ or RRAD adenovirus for 48 h. RRAD transduction in hCOs resulted in a slight increase in RRAD mRNA expression due to inefficient tissue penetration of the adenovirus **(Supplementary Figure S5A)**. Nevertheless, even at low levels of RRAD overexpression, we observed a significant increase (~15%) in CM nuclei number **(Supplementary Figure S5B)** and a trend to increase PHH3 positive CM nuclei **(Supplementary Figure S5C)**. Functionally, hCOs only had a modest (~4%) reduction intracellular Ca^2+^ amplitude and a decrease in the force of contraction of ~6%. (**Supplementary Figure S5D-E**). These functional changes indicate that the induction of cell cycle activity through RRAD overexpression can be supported while the cardiac tissue remains functional.

### RRAD overexpression promotes complete CM cell division and primes more CMs to proliferate in response to cell cycle stimuli in vivo.

**Figure 5:**
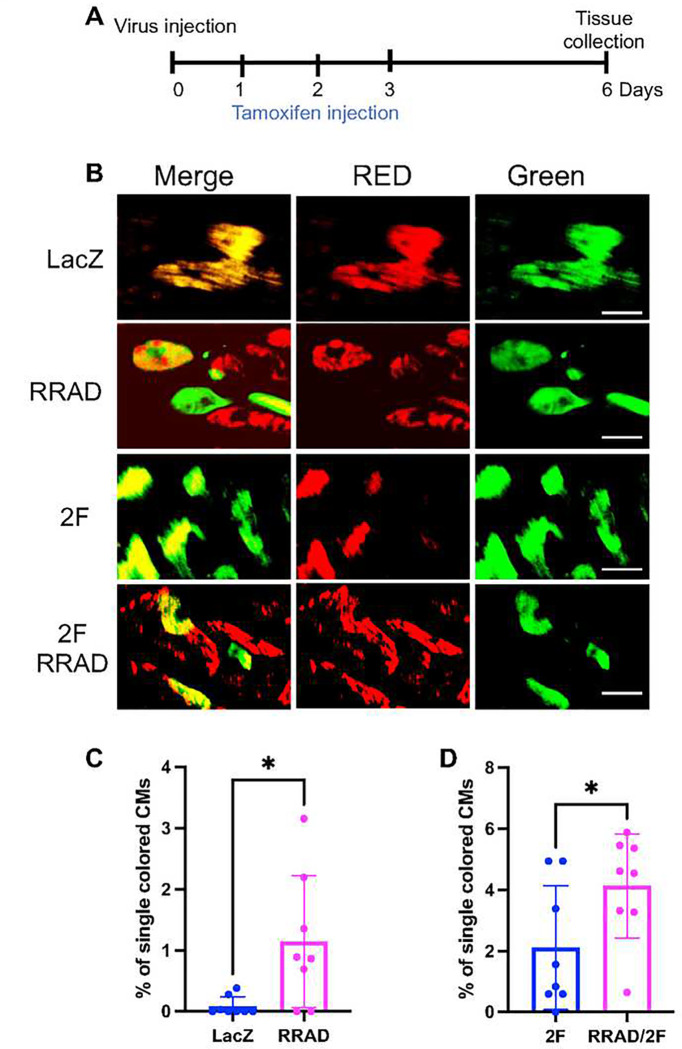
Complete CM division occurs in MADM mice following RRAD overexpression. (A) Schematic diagram of the experimental design for MADM mice injection with LacZ, RRAD, CDK4/CCND (2F), or RRAD/2F. **(B)** Representative images show single-colored CMs of MADM mice hearts treated with LacZ, RRAD, CDK4/CCND (2F), or RRAD/2F (Scale bar=20μm). **(C)** Quantification of the percentage of the single-colored CMs to the total labeled CMs (n=8). **(D)** Quantification of the percentage of single-colored CMs to the total labeled CMs after 2F or 2F+RRAD overexpression Data are presented as mean ± SD,. *p<0.05.

To investigate the effect of RRAD overexpression on CM proliferation and complete cytokinesis in vivo, a cardiomyocyte cytokinesis lineage-tracing animal model (inducible a-MHC-Cre::MADM-lineage-tracing) (MADM mice) ^19,20,23–25^ was used. In these lineage-tracing mice, CMs that undergo cytokinesis produce daughter cells that are either red, green, yellow (red+green), or colorless, based on allelic recombination of fluorescent reporters; if the CMs fail to divide, they will remain double-colored (i.e., yellow), or colorless if no recombination occurs. Thus, the presence of single-colored red or green cells is a definitive indicator that these CMs have undergone complete cytokinesis. However, dividing CMs are underrepresented by single-colored CMs because double-colored (yellow) or colorless cells also could have divided. MADM mice were injected with either LacZ, RRAD, 2F, or RRAD+2F adenoviruses (intramyocardially). Tamoxifen injection was carried out as described in ^20^, starting 24 h after the virus injection for 3 days to initiate recombination events. Mice were sacrificed 6 days after the viral infections, and hearts were sectioned to enumerate the cytokinesis events (Fig. 5A). RRAD overexpression was confirmed by RT-PCR **(Supplementary Figure S4B)**. Mice hearts in which RRAD was overexpressed showed a significant increase in the fraction of single-colored CMs compared to hearts injected with LacZ control virus (Fig. 5B, C). Moreover, RRAD overexpression in combination with 2F primed more CMs to proliferate, as indicated by a significant increase in single-colored CMs compared to 2F-treated hearts (Fig. 5B, D). These data demonstrate that RRAD overexpression promotes CM proliferation in vivo.

### Mechanistically, RRAD overexpression modulates calcineurin activity, which induces cell cycle activity in CMs.

**Figure 6:**
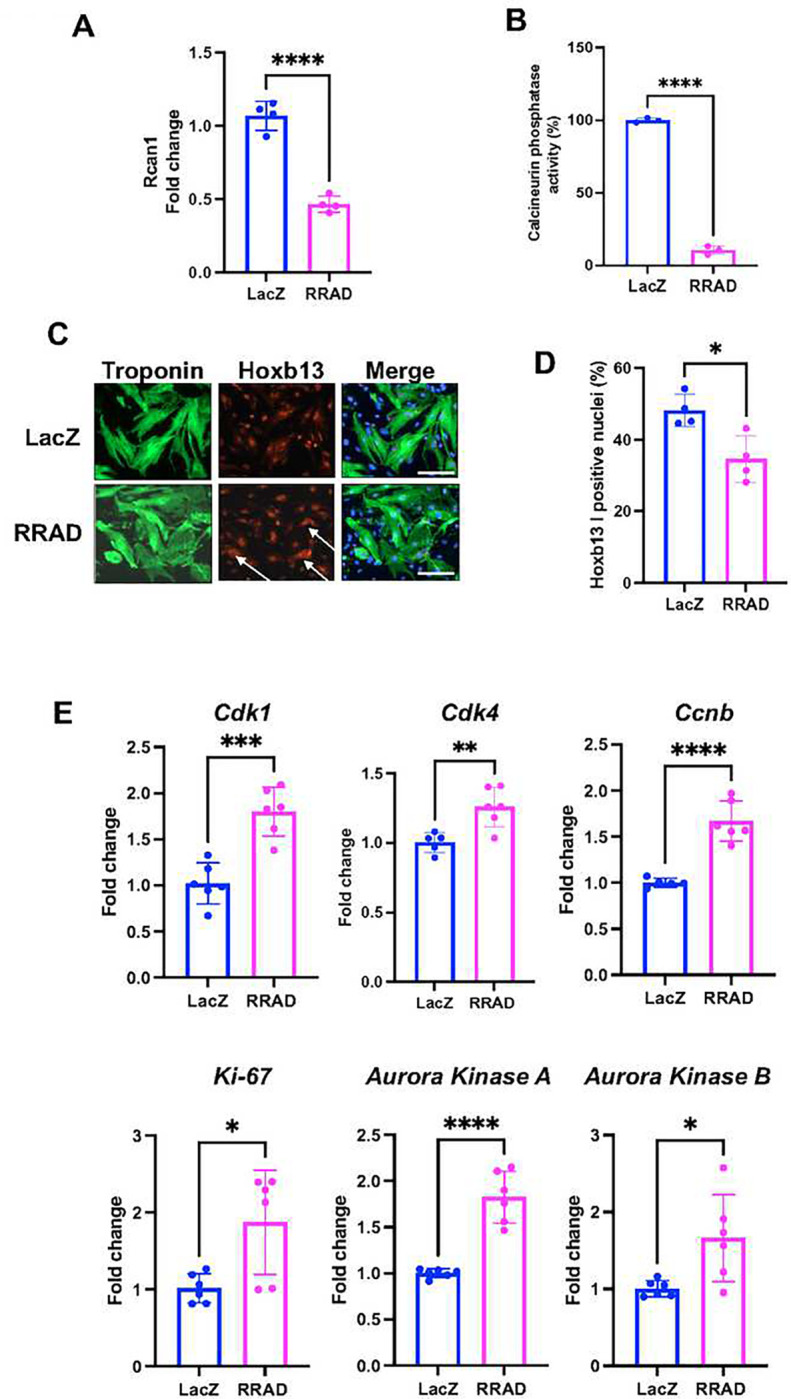
Following RRAD overexpression, calcineurin activity was reduced, and cell cycle gene expression was increased. NMCM P7 transduced with LacZ or RRAD adenovirus for 72 h. **(A)** Quantification of the calcineurin phosphatase activity (n=3). **(B)** Fold change analysis of the mRNA expression of (Rcan1) (n=4). Immunostaining against troponin-T (Green), DAPI (Blue), **(C)** Hoxb13 (Red) (scale bar 100μm). Arrows pointing to Hoxb13 in the cytoplasm only. **(D)** Bar graph for percentage Hoxb13 positive CM nuclei (n=5) **(E)** Fold change of cell cycle gene expression in NMCM P7 transduced with LacZ or RRAD Adenovirus for 72 h **(**n=6). Data are presented as mean ± SD, *p<0.05, **p<0.01, ***p<0.001, ****p<0.0001.

**Figure 7:**
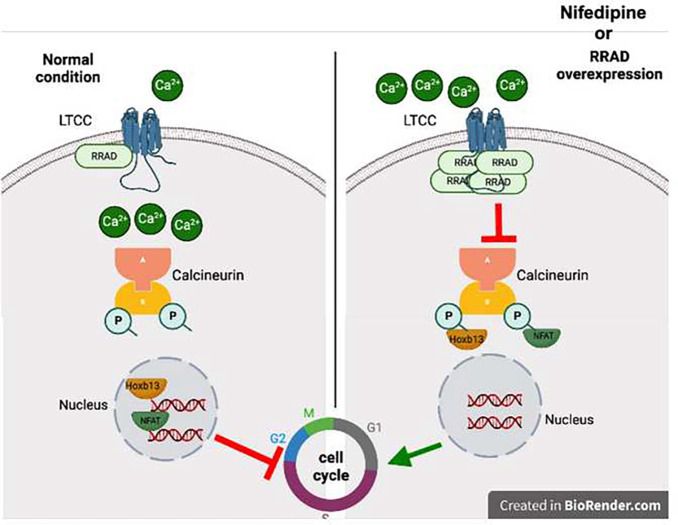
Schematic diagram of the mechanism by which RRAD promotes CM proliferation.

Calcineurin is a Ca^2+^-dependent phosphatase enzyme localized in the plasma membrane in close proximity to LTCC ^14–17^. Recently, reduced calcineurin activity was reported to induce CM cell cycle activity ^7,13^. To investigate the effect of RRAD overexpression on calcineurin activity, P7 NMCM were infected with LacZ or RRAD adenovirus for 72 h and examined for cellular calcineurin phosphatase activity. RRAD overexpression significantly reduced calcineurin phosphatase activity compared to LacZ control (Fig. 6A). The regulator of calcineurin (Rcan1) is an endogenous regulator of calcineurin activity. Expression of the Rcan1 is under calcineurin control and thus functions as a feedback inhibitor of calcineurin, and its expression is an indicator of calcineurin activity ^26^. In P7 NMCM, RRAD overexpression significantly reduced Rcan1 mRNA expression compared to LacZ control (Fig 6B). Hoxb13 are downstream transcription factors dephosphorylated by calcineurin and then translocated to the nucleus to inhibit the cell cycle in CMs^7^. We observed significantly reduce Hoxb13 nuclear localization in P7 NMCM overexpressing RRAD (Fig. 6C–F). In support of their role in cell cycle regulation, RRAD overexpressing P7 NMCM showed a significant increase in the cell cycle gene activators (Fig. 6G). These data suggest that inhibition of LTCC via overexpression of RRAD reduces calcineurin activity, which subsequently attenuates Hoxb13 translocation to the nucleus and hence induces expression of cell cycle gene activators and CM proliferation (Fig 7).

### Bulk RNA sequencing demonstrates that LTCC inhibition for 8 h actively regulates CM structural genes.

To investigate the early transcriptional changes associated with LTCC inhibition, we conducted bulk RNAseq on hCOs treated with vehicle or nifedipine for 8 h (5 individual hCOs per condition). Comparing the global gene expression, nifedipine-treated hCOs showed over 650 genes that were differentially expressed, of which 442 genes were down-regulated, and 219 genes were up-regulated (FDR <0.05) **(Supplementary Figure S6A & Source Data 1)**. The GO terms analysis reflects that the differentially regulated genes are mostly involved in cardiac function and sarcomere integrity **(Supplementary Figure S6B-C).** Eleven of the top downregulated genes in nifedipine treated hCO were reported to be downstream transcriptional targets for Hoxb13/Meis1^7^**(Supplementary table 3, Source Data1).** These data demonstrate that the early transcriptional changes following inhibition of LTCC are likely to be due to inhibition of Hoxb13/Meis1 DNA binding as a downstream effect of calcineurin inhibition in CMs.

## Discussion

Activating the CM cell cycle to regenerate functional myocardium remains an aspirational goal. Understanding the molecular mechanisms controlling the CM cell cycle may unlock new strategies to achieve this goal. We and others have shown that contractility precludes cell cycle induction in CMs ^19,27–29^, but the underlying mechanism is not clear. Ca^2+^ is a critical regulator of CM contractility and also plays a vital role in other cellular processes, including cell adhesion, gene transcription, metabolism, and survival ^3–7^. Our previous study demonstrated that genes involved in CM contractility and Ca^2+^ cycling are downregulated during forced cell cycle induction using 4F ^19^, suggesting an association between these processes. However, we and others have not yet defined the mechanisms behind this relationship. Our current study suggests that inhibition of Ca^2+^ influx through LTCC promotes cell cycle induction by deactivating calcineurin activity, which enhances cell cycle gene expression in CMs. Thus, inhibition of LTCC could be a putative target for developing new therapies to induce CM proliferation and regeneration.

Here, we demonstrate that reducing Ca^2+^ influx through inhibition of LTCC (nifedipine) or reducing the Ca^2+^ concentration in the media attenuates CM contractile function. Yet only nifedipine induced the CM cell cycle. These results are in line with a recent study that screened several drugs to assess the proliferation of human-induced pluripotent stem cell-derived CMs. They showed that LTCC blockers from multiple chemical classes are among the most potent drivers of proliferation ^8^. Another study also demonstrated that the inactivation of the β-AR signaling, an upstream regulator of LTCC activity, increases CM division in neonatal mice ^28^. Thus, the LTCC itself seems to be an important regulator of the response.

In our study we have uncovered how LTCC activity controls proliferation. Our single-cell RNAseq data from P1 NMCM demonstrated that RRAD, which is an endogenous inhibitor of LTCC, is highly expressed in spontaneously proliferating and primed CM populations. RRAD was originally identified in the skeletal muscle of patients with type 2 diabetes mellitus ^30,31^. RRAD in CMs binds directly to LTCC β -subunits and controls LTCC current (I_Ca, L_) in CMs ^10,11^. Modulation of RRAD expression and its impact on cardiac Ca^2+^ handling has been extensively studied. Deficiency of RRAD function in CMs leads to an increased I_Ca, L_^32–34^. Dominant negative suppression of RRAD (S105N) led to an increase in I_Ca, L_ and prolonged the action potential via upregulation of LTCC channel expression in the plasma membrane of guinea pig ventricular CMs ^35^. Moreover, RRAD cardiac-specific KO hearts exhibited enhanced cytosolic Ca^2+^ flux, increased contractile function, elevated sarcoplasmic/endoplasmic reticulum calcium ATPase 2 (SERCA2a) expression, and faster lusitropy ^32^. In contrast, RRAD overexpression inhibits the I_Ca,L_ in adult and embryonic ventricular myocytes ^11,12^. RRAD has also been involved in regulating Ca^2+^ dependent signaling in the heart where it modulates cardiac hypertrophy through modulation of the calcium-calmodulin-dependent protein kinase II (CAMKII) ^36^. Moreover, a recent study reports that RRAD expression is increased at the border zone following myocardial infarction in human hearts ^37^ while other studies reported that RRAD protein levels fall in patients with end-stage non-ischemic heart failure (heart failure with reduced ejection fraction, HFrEF) ^33^, and in a mouse model of cardiac hypertrophy ^36^. RRAD thus plays an integral role in cardiac signaling and homeostasis.

The small molecule screen as well as the single cell transcriptomics data both suggested the role of LTCC-RRAD in regulating cell cycle induction. However, to avoid potential off-target effects of a small molecule ^38–41^, we preferred to continue studying the role of LTCC in cell cycle induction through manipulating its activity using RRAD overexpression. The direct genetic manipulation of LTCC was not possible as genetic deletions of the LTCC α1C or the β2 encoding genes result in embryonic lethality with heart dysfunction ^42,43^. In addition, genetic ablation of the LTCC complex may disrupt the function of proteins that physically interact with LTCC and result in an off-target effect ^44–46^. Therefore, overexpression of RRAD, which explicitly inhibits I_Ca, L_, enabled validation of the role of Ca^2+^ influx through LTCC on CM cell cycle induction. Given that CMs are prone to undergo binucleation, we specifically studied late cell cycle stages, including G2-M and cytokinesis, to confirm complete cell division. We used PHH3 expression as a marker for the G2-M phase and Aurora Kinase B to identify CMs that entered cytokinesis. RRAD overexpression in vitro (P7 NMCM) and ex-vivo (human heart slices) demonstrated that RRAD indeed promotes CM proliferation through mitosis and cytokinesis. Using MADM mice hearts, we demonstrated that RRAD overexpression induces complete CM cytokinesis and cell division in vivo as well. MADM mice are α-MHC-Cre::MADM-lineage-tracing mice that are widely used to prove complete cell division in vivo ^19,20,23–25^. RRAD adenovirus was injected directly into the heart at a minimum dose, and no mortality was observed during the current experiment. The data obtained with hCOs provided further evidence that CM cell cycle activity can occur while maintaining minimal functional changes. Therefore, mild inhibition of the I_Ca, L_ through RRAD overexpression promotes CM proliferation to similar levels as nifedipine.

Ca^+2^ influx through LTCC regulates several signaling pathways, including calcineurin activity ^14–17,45,47^. We observed reduced calcineurin activity and nuclear localization of Hoxb13 following RRAD overexpression. It was recently reported that calcineurin is colocalized and interacts with LTCC ^48^ and that calcineurin plays a role in CM cell cycle arrest ^7,13^. Inducible CM-specific deletion of calcineurin B1 in adult CMs promotes mitotic entry, improves cardiac function, and reduces scar size after myocardial infarction. Moreover, a small molecule inhibitor for calcineurin, FK506, promotes CM proliferation. However, FK506 fails to improve cardiac function or reduce scar size after MI due to inhibition of vasculogenesis and blunting of the post-MI inflammatory response ^13^. Calcineurin acts by dephosphorylating Hoxb13 at serine-204 causing translocation to the nucleus and leads to cell cycle arrest. Expression of both calcineurin and Hoxb13 are significantly higher in p7 postnatal CMs, which coincides with cell cycle arrest during development. These reported data, in combination with our current study, further establish the role of the calcineurin/Hoxb13 axis in regulating CM cell cycle activity.

Hoxb13 acts as a cofactor of Meis1, which is a three amino acid loop extension (TALE) family homeodomain transcription factor. Meis1 translocates to CM nuclei shortly after birth and mediates postnatal cell cycle arrest. Adult Meis1-Hoxb13 double-knockout hearts showed a significant increase in the CM mitosis and sarcomere disassembly, which improved left ventricular systolic function following myocardial infarction^7^. Our bulk RNA sequencing investigating the early transcriptional changes associated with LTCC inhibition by nifedipine also suggested sarcomere remodeling. We observed downregulation of genes responsible for cardiac structure, integrity, and sarcomere assembly, many of which are downstream transcriptional targets for Hoxb13/Meis1^7^ **(Supplementary table S4).** This suggested that the early transcriptional changes post LTCC inhibition were associated with downregulation of Hoxb13/Meis1 transcriptional program in CMs.

In conclusion, we demonstrate that pharmacological or genetic inhibition of Ca^+2^ influx through LTCC is an upstream regulator of the calcineurin/Hoxb13 axis and stimulates cell cycle activity in CMs in vitro and in vivo.

### Limitation to the study:

One of the major limitations of altering intracellular Ca^2+^ levels is the clinical applicability of such an approach in treating ischemic heart failure in humans. Future work is needed to examine the effect of RRAD overexpression or a cocktail of RRAD and other cell cycle stimulators on cardiac function and scar size after myocardial infarction. This will first need extensive optimization of a dose of RRAD overexpression that transiently inhibits the LTCC current without a major effect on the contractile function while simultaneously inducing CM proliferation for a short time. This could potentially be achieved through modified RNA delivery, which would be transient and dose-controlled.

## STAR Methods

### Human embryonic stem cells:

Ethics approval for the use of the human embryonic stem cell (hESC) line Hes3 (female) (WiCell) was obtained from QIMR Berghofer’s Ethics Committee (2014000801 and P2385). All work was performed per the National Health and Medical Research Council of Australia (NHMRC) regulations. hESCs were maintained on Matrigel-coated flasks (Cat#NUNC156367, Corning) in mTesR+ ([Cat#05825, Stem Cell Technologies) and passaged every 2–3 days using RelesR (Cat#05872, Stem Cell Technologies).

### Cardiac differentiation:

hESC was differentiated into cardiac cell types using previously published protocols^18,49–53^. This differentiation process generates multi-cellular cultures consisting of CMs (~70%) and stromal cells (~30%). hESC (seeded at 2×10^4^ cells/cm^2^) were prepared for differentiation by culturing in mTeSR-1 medium (Stem Cell Technologies) for 4 days. Differentiation into cardiac mesoderm was initiated by changing the culture media to RPMI B27- medium (RPMI1640 GlutaMAX (Cat#61870-036) + 2% B27 supplement without insulin (Cat#A1895601), 200 mM L-ascorbic acid 2-phosphate sesquimagnesium salt hydrate (Cat#A8960-5G, Sigma) and 1% Penicillin/ Streptomycin (Cat#10378-016) (all ThermoFisher Scientific unless otherwise indicated)) containing 5 ng/mL BMP-4 (Cat#RDS314BP050CF, RnD Systems), 9 ng/mL Activin A (Cat#RDS388AC050, RnD Systems), 5 ng/mL FGF-2 (Cat#RDS233FB025, RnD Systems) and 1 mM CHIR99021 (Cat#73042, Stem Cell Technologies). Media was changed daily for 3 days, after which specification into a hESC-CM/stromal cell mixture using RPMI B27- containing 5 mM IWP-4 (Cat#72554, Stem Cell Technologies) was induced. The next day, the medium was changed to RPMI B27+ medium (RPMI1640 GlutaMAX + 2% B27 supplement with insulin (Cat#17504044), 200 mM L-ascorbic acid 2-phosphate sesquimagnesium salt hydrate and 1% Penicillin/Streptomycin) with medium exchange every 2–3 days for 7 days. The differentiated cells were then cultured in RPMI B27+ medium until harvest on day 15 of the differentiation protocol. Cardiac cell types were released from the culture flask using 0.2% collagenase type I (Cat# C0130, Sigma) in 20% fetal bovine serum (FBS) in PBS (with Ca2+ and Mg2+) for 60 min at 37°C, followed by 10 min in 0.25% trypsin-EDTA (Cat# 2500–072, ThermoFisher Scientific). Deactivation of trypsin-EDTA was performed using a-MEM GlutaMAX (Cat# 32561-037, ThermoFischer Scientifi), 10% FBS (Cat#10099-141, ThermoFischer Scientific), 200 mM L-ascorbic acid 2-phosphate sesquimagnesium salt hydrate, and 1% Penicillin/Streptomycin at equal volumes. Cells were then filtered using a 100 mm mesh cell strainer (BD Biosciences) and counted using a hemocytometer.

### Heart Dyno hCO production:

To produce hCOs, the cardiac cell types were seeded into heart dyno culture inserts^18^. These inserts were produced using standard SU-8 photolithography and PDMS molding practices. Each insert, with two PDMS poles that guide hCO formation, was glued into a well of a 96-well plate. hCOs were produced by mixing 5×10^5^ cells in base medium (a-MEM GlutaMAX (ThermoFisher Scientific), 10% fetal bovine serum (FBS) (ThermoFisher Scientific), 200 mM L-ascorbic acid 2-phosphate sesquimagnesium salt hydrate (Sigma) and 1% Penicillin/Streptomycin (ThermoFisher Scientific)) with bovine acid-solubilized collagen I (Cat# 01PA006, Devro, 2.6 mg/mL final concentration), which was salt balanced and pH neutralized with 10X DMEM (Cat#12100046, ThermoFisher Scientific) and 0.1M NaOH, respectively, and Matrigel (Cat#FAL354277, 9% final concentration). The mixture and culture plate were kept on ice while 3.5 μL was seeded into each heart dyno insert, which had been coated with 3% bovine serum albumin (Cat#A9418, Sigma) in PBS. The 96-well plate was centrifuged for 10 sec at 100×g before placing the plate in the incubator (37°C, 5% CO_2_) for ~45 min to let the mixture gel. Subsequently, 150 μL base medium was added, and hCOs were cultured for 2 days, during which hCO condensation occurred. The medium was then changed to maturation medium (MM), which consists of DMEM without glucose, glutamine, and phenol red (Cat# A11430-01, ThermoFisher Scientific) supplemented with 4% B27- (without insulin) (ThermoFisher Scientific), 1% GlutaMAX (Cat# 3505–061, ThermoFisher Scientific), 200 mM L-ascorbic acid 2-phosphate sesquimagnesium salt hydrate and 1% Penicillin/Streptomycin (ThermoFisher Scientific), 1 mmol/L glucose and 100 mmol/L palmitic acid (Cat# P0500, Sigma) (conjugated to bovine serum albumin within B27 by incubating for 2h at 37°C) (media change every 2–3 days) to induce hCO maturation through induction of a metabolic switch to oxidative metabolism ^18^. Following 7 days of maturation, the medium was changed to weaning medium (WM) (4% B27- insulin, 5.5 mM glucose, 1nM insulin (Cat#12585014, Sigma), 200 mM L-ascorbic acid 2-phosphate sesquimagnesium salt hydrate, 1% penicillin/streptomycin, 1% GlutaMAX (100X), 33mg/mL aprotinin (Cat# HY-P0017-500MG) and 10 mM palmitate (conjugated to bovine serum albumin in B27) in DMEM without glucose, glutamine and phenol red) to mimic the mature metabolic environment of the heart *in vivo*^53^. hCOs were cultured in WM for at least 7 days with media changes every 2–3 days until experimental treatments. All experimental treatments were performed for 48h in WM, after which hCOs were fixed.

#### Adenoviral transduction and drug intervention in hCOs

hCOs were transduced with LacZ and RRAD adenovirus at 50 MOI. The culture medium was refreshed to WM without adenovirus 24 h post-infection. Nifedipine (Sigma # N7634), thapsigargin (Sigma # T9033), and ryanodine (Abcam # ab120083) were dissolved in DMSO and then diluted to the required concentration in WM medium.

### Functional analysis:

Live videos of 10 seconds (50 frames per second) were obtained using the Leica Thunder microscope with climate control (37°C, 5% CO_2_). From these videos, the deflection of the PDMS poles in the heart dyno insert can be measured using a custom-written MatLab script (MATLAB R2013a (Mathworks))^18^ which provides readouts of multiple functional parameters, including force, rate, activation, and relaxation time.

### Assessing intracellular calcium amplitude in hCO:

After 48 h of adenovirus overexpression, hCOs were incubated with WM supplemented with Fluo-4 AM (Thermo Fisher Scientific, F14201) for 60 min at 37°C, 5% CO_2_. Subsequently, the medium was changed to WM without Fluo-4 AM, and the hCOs were incubated for another 30 min at 37°C, 5% CO_2_ before imaging. Videos (10 sec) were obtained using the Leica Thunder (magnification 5x, exposure 25ms, laser 470 nm, filter 510 nm). Analysis was performed using a custom-written MatLab script (MATLAB R2013a (Mathworks)). Three regions of interest were selected for each hCO, and the mean F/F0 was determined.

### qRT-PCR on hCOs:

Following treatment, hCOs were washed with ice-cold PBS, removed from the Heart Dyno insert, and pooled per condition (4–8 hCOs). They were then resuspended in RLT buffer supplemented with 10 μL/mL β-mercaptoethanol (Sigma) and snap-frozen at −80 °C until use. RNA was extracted using the Qiagen RNeasy micro kit (74004) as per manufacturers’ recommendations. RNA concentration was determined using the Nanodrop 2000 (Thermo Fisher Scientific). Primer annealing was done using Random Primers (58875, Invitrogen, 3 μg/μL) and dNTP mix (Invitrogen, 10 mM) (incubation 5 min at 65°C followed by 5 min at 4°C). cDNA master mix was prepared using Superscript III Reverse Transcriptase (56575, Invitrogen, 200u/μL), RNase Out (100000840, Invitrogen, 40u/ μL), 5x First Strand Buffer (Y02321, Invitrogen), and DTT (Y00147, Invitrogen, 0.1M). The samples were incubated for 3 min at 25 °C, 60 min at 50 °C, and 15 min at 70 °C. qPCR was run using PowerUp SYBR Green Master Mix (A25742, Applied Biosystems) on the ABI Quantstudio 5 QPCR machine (Thermo Fisher Scientific). Data was analyzed using the 2^−ΔΔCt^ method to determine changes in gene expression with 18S as the housekeeping gene. Primers **(Supplementary Table S1)** were used at 200 nM.

### Immunocytochemistry for hCOs

hCOs were fixed in 1% paraformaldehyde for 1 h at room temperature and washed 3x with PBS. To stain the hCOs, they were incubated in blocking buffer (5% FBS and 0.2% Triton X-100 (T8787, Sigma) in PBS) for at least 2 h at 4°C on a rocker. The hCOs were then incubated with the primary antibodies **(Supplementary Table S2)** overnight at 4°C on a rocker. The next day, hCOs were washed with blocking buffer 2x for at least 2 h at 4°C on a rocker before incubating with secondary antibodies **(Supplementary Table S2)** overnight at 4°C on a rocker. hCOs were rewashed in blocking buffer (2X) for at least 2 h before imaging. hCOs were imaged while still in the heart dyno plate in either PBS or fructose–glycerol clearing solution (60% (vol/vol) glycerol (G2025, Sigma) and 2.5 M fructose (F0127, Sigma)). Whole hCO images were obtained using the Leica Thunder (5x or 20x magnification). For higher magnification, hCOs were mounted on slides in ProLong Glass mounting media (P36980, ThermoFisher Scientific). The Zeiss 780-NLO Point Scanning Confocal Microscope was used to obtain detailed images at 63x magnification. For initial screening purposes, whole hCO intensity was measured using custom batch processing files in MATLAB R2013a (Mathworks) to remove the background, calculate the image intensity, and export the batch data to an Excel (Microsoft) spreadsheet. To quantify Ki-67 or pHH3 positive CM nuclei, three random images were obtained per hCO. Ki-67 positive or PHH3 positive CM cells were manually quantified by counting the total number of nuclei that co-localized with α-actinin and the total number of Ki-67 or PHH3 positive nuclei. The numbers of the three images per tissue were combined to calculate the percentage of Ki-67 or PHH3 positive nuclei per hCO. Quantification of the number of CM nuclei (NKX2.5+) in hCOs was done on 6 merged 20X Tilescan images. These images were analyzed using a custom-built Strataquest application (version 7.1.1.129) from TissueGnostics.

### Bulk RNA Sequencing

#### RNA extraction and library preparation

hCOs were treated with nifedipine or DMSO (vehicle control) for 8 hours (n = 5 hCOs per condition). hCOs were washed with PBS, lysed, and stored at −80 degrees. RNA extraction was conducted using the NucleoMag 96 RNA kit (Macherey-Nagel) on the EpMotion 5075t. RNA was quantified using the Qubit RNA High Sensitivity Assay (Invitrogen). RNA integrity was assessed using the 4200 TapeStation with the High Sensitivity RNA ScreenTape kit (Agilent). Samples were normalized to ≤100 ng RNA using nuclease free water in a total of 25μl. Poly(A)-enriched RNA libraries were prepared using the Illumina Stranded mRNA Prep, Ligation (96 Samples), and the IDT for Illumina RNA UD Indexes Set A (Integrated DNA Technologies). Libraries were quantified using the Quant-iT dsDNA broad range (BR) assay kit (Invitrogen). The quality of libraries was assessed using the 4200 TapeStation with the D1000 ScreenTape kit (Agilent). Single-end 100bp sequencing was conducted on the NovaSeq 6000 (Illumina).

#### Read mapping and quantification.

Basecalling and adapter trimming was completed on BaseSpace (Illumina). ‘FastQC’ (version 0.11.9) was run for quality control. Reads were aligned using ‘STAR’ ^54^ (version 2.7.9a) to the GRCh38 assembly with the gene, transcript, and exon features of the Ensembl (release 106) gene model. BAM files for each sample were merged using samtools (version 1.9) ^55^. RSEM ^56^(version 1.3.1) was used to quantify expression. Duplicate reads were marked using Picard MarkDuplicates in the GATK package (version 4.2.4.1). `RNA-SeQC`^57^ (version 2.4.2) was used to compute quality control metrics. Org.Hs.eg.db (version 3.14.0) was used to annotate gene biotypes. All gene biotypes were kept for further analysis. GO term analysis was conducted using the ‘TF perturbations followed by expression’ database on https://maayanlab.cloud/Enrichr/.

#### Differential Expression Analysis

Differential expression analyses were performed using edgeR ^58^ (version 3.36.0) using the glmQLFit() and glmQLFTest() functions. Multiple testing correction was performed by applying the Benjamini-Hochberg method on the p-values to control the false discovery rate (FDR). Differentially expressed genes (DEGs) were determined using the cut-off FDR < 0.05. Volcano plots were generated using EnhancedVolcano (version 1.12.0) https://bioconductor.org/packages/devel/bioc/vignettes/EnhancedVolcano/inst/doc/EnhancedVol cano.html. clusterProfiler (version 4.2.2) ^59^ was used for enrichment analysis of gene ontology (GO) terms, using the enrich GO function. Adjusted p-values were calculated by the BH method, and results were visualized using the dotplot function from enrichplot (version 1.14.2). https://yulab-smu.top/biomedical-knowledge-mining-book/

#### Preparation of P1 or P7 neonatal cardiomyocytes

Primary mouse cardiomyocytes were isolated from 1 day or 7-day-old C57/Bl6 mice. The P1 or P7 neonatal cardiomyocytes were isolated by the established protocol described in ^20,60^. Briefly, neonatal pups were sacrificed, and hearts were isolated. The ventricular tissue was cut into small pieces and was digested by several rounds of 7-minute incubations in ADS buffer (0.68% NaCl (w/v), 0.476% HEPES (w/v), 0.012% NaH2PO4 (w/v), 0.1% glucose (w/v), 0.04% KCl (w/v), 0.01% MgSO4 (w/v), pH 7.35) containing 0.3 mg/ml collagenase A (Roche) and 0.6 mg/ml pancreatin (Sigma-Aldrich) at 37°C with continuous shaking. 3ml of Fetus bovine serum (FBS, Invitrogen) was added to each digestion. All the digested samples were pooled together, and cells were centrifuged and resuspended in a plating medium (68% DMEM, 17% medium 199, 10% horse serum, 5% FBS) (Invitrogen). CMs were separated from non-CM cells by plating the mixture on tissue culture plates for 1h at 37°C in 5% CO_2_. The suspended CMs were then plated on laminin-coated tissue culture plates and incubated overnight at 37°C in 5% CO_2_ to allow cellular attachment. The following day, cells were washed with PBS, and the plating medium was replaced with maintenance medium (79.5% DMEM, 19.5% medium 199, 1% FBS (Invitrogen)) supplemented with 1% Antibiotic-Antimycotic (Invitrogen).

##### Adenoviral transduction and drug Intervention in NMCM P7:

NMCM P7 were transduced with adenovirus at the following MOI: LacZ and RRAD 1 MOI, 4F (CDK1AF, CCNB1, CDK4, CCND1), 2F (CDK4, CCND1), CCNA2 10 MOI each (vector biolabs); Adenoviruses were removed after 24 h and replaced with maintenance medium. Nifedipine (Sigma-Aldrich, N7634) was diluted to 1 mM in DMSO and then diluted to the required concentration in maintenance media.

### Single-Cell RNA-Sequencing Library Generation

NMCM P1 was isolated as described above and transduced with LacZ or 4F for 24 h. The CM were then trypsinized and filtered using a 70μM filter, resuspended in 1% B.S.A. in PBS, and counted immediately before library preparation. G.E.M. Generation and Barcoding were performed following the Chromium Single Cell 3’ Reagents Kits v3 Rev A User Guide with Chromium Single Cell” G.E.M., Library, and Gel Bead Kit v3 (10X Cat # 1000092). Briefly, the Chromium chip (Cat # 1000074) was loaded, and the Chromium Single Cell B program was selected. The chip was ejected immediately following the completion of the program. 100 μL of recovered G.E.M.s was slowly pipetted and transferred to tubes pre-cooled on ice. The G.E.M.s were then incubated for the R.T. reaction using a thermal cycler at 53°C for 45 min, then 85°C for 5 min, followed by a 4°C hold. Samples were then stored at −20°C overnight. All libraries were pooled and sequenced using the NovaSeq to a read depth of at least 50,000 reads per cell. The sequenced reads for each sample were mapped to the GRCh38 genome to generate the (gene-by-cell) count matrix using cell ranger count (Cell Ranger version 3.1.0 from 10x Genomics) with default parameters. The counts” matrices across the samples were aggregated using cellranger-aggr. The resulting files were processed in R using the package Seurat (version 3.1.3)^61^. All cells with at least 200 detected genes and less than 30% of reads from mitochondrial genes and all detected genes in at least 3 cells were used in the further analyses. The remaining data were normalized using the “LogNormaliz” method. Principal Component Analysis for the subset of the 2000 most variable genes (Seurat function FindVariableFeatures) was then performed on the scaled data. The cells were clustered using the Louvain Algorithm with the resolution parameter value of 0.5 (Seurat function FindClusters) after determining the shared nearest neighbor graph using the first ten principal components (Seurat function FindNeighbors). The data were visualized using the UMAP algorithm with the first ten principal components as input (Seurat function RunUMAP). The subpopulations were labelled based on the distribution of expression of the cell cycle genes of interest. Differential analysis between all pairs of clusters was performed using the Wilcoxon rank-sum test to identify the differentially expressed genes (Seurat function FindMarkers).

#### Immunocytochemistry and EdU incorporation in NMCM P7/ heart slices:

NMCM were fixed in 4% formaldehyde for 20 min (Thermos Scientific Cat#28908). For human heart tissue, heart slices were fixed in 4% paraformaldehyde for 48 h. Fixed tissue was dehydrated in 10% sucrose for 1 h, followed by 20% sucrose for 1 h, and kept overnight in 30% sucrose at 4°C. The dehydrated tissue was then processed in optimal cutting temperature compound (OCT compound) and gradually frozen in an isopentane/dry ice bath. OCT-embedded blocks were stored at −80°C for 24 h. Sections (8 μm) were cut using a cryostat (Leica Inc.) and stored at −20°C. To remove the OCT compound, the slides were heated for 5 min at 95°C until the OCT compound melted. Slides were washed with 1 mL of PBS and incubated at RT for 15 min until the OCT compound was washed off. Fixed cells or tissue were washed three times with PBS. Next, the fixed cells/ heart slices were permeabilized with 0.1% Triton X-100 for 15 min (Millipore Cat# 55163804) and then blocked with 3% bovine serum albumin (BSA) in PBS for 60 min at RT (VWR Cat# 0332). The cells/ heart slices were then probed with primary antibody for 1.5 h **(Supplementary Table S3)**. They were then washed three times with PBS and labeled with a secondary fluorescent antibody **(Supplementary Table S3).** Next, cells/tissue were washed three times with PBS and stained with DAPI 1μg/ml (Biotium Cat# 40043) to stain the nucleus. For EDU detection, the cells were also treated with 5μM 5-ethyl-2-deoxyuridine (EDU) for the course of the experiment, which will be incorporated into the newly synthesized DNA. After fixation, permeabilization, and blocking of the cells, the EDU incorporation was visualized using the Click it EDU-Alexa-Flour^647^ imaging kit (ThermoFisher, C10340). Imaging was conducted for the whole well (15000 cells per well in black-walled, glass bottom 96 well) using the high-content imaging instrument, Cytation 1. The percentage of colocalization of PHH3, EDU,Hoxb13 on nuclei and Troponin-T was quantified using Gen 5.05 software. Aurora Kinase B staining was imaged at 10X magnification using the high-content imaging instrument Keyence and was quantified using BZX800 software.

#### qRT-PCR in NMCM P7/ heart slices/ heart tissue:

NMCM P7, human heart slices, or heart tissue were mixed with QlAzol lysis reagent (Qiagen Cat# 79306), then RNA was extracted following the miRNeasy micro kit protocol (Qiagen Cat# 217084). The concentration of the RNA was calculated using the Cytation 1 reader. 0.2ug of each RNA sample was used for reverse transcription using a mixture of oligo(dT) and random hexamer primers (SuperScript IV VILO Master Mix, ThermoFisher Scientific Cat # 11756050). Real-time PCR analysis was conducted with TaqMan fast advanced master mix (ThermoFisher 4444557) and primers specific to mouse CDK1 (Thermofisher # 00772472), mouse CDK4 (Thermofisher # 00677718), mouse CCNB (Thermofisher # 03053893) mouse Ki-67 (Thermofisher # 01278617), mouse Arura A (Thermofisher # 01248177), mouse Arura B (Thermofisher # Mm01718146), mouse Rcan1 (Thermofisher # 01213406) and the expression was normalized to mouse GAPDH expression (Thermofisher # 99999915) using the Quant studio5 real-time PCR detection system (Applied Biosystems).

### Western blot

Cells were lysed in RIPA buffer (VWR, N653) in the presence of a Protease and Phosphatase Inhibitor Cocktail (Thermofisher Scientific, 78440). The lysate was denatured at 95°C for 5 min, after which they were loaded and separated on 4–12% Bis-Tris gel (Invitrogen, NW64120) and subsequently transferred onto PVDF membranes (Thermofisher scientific, # LC2002). The membrane was then blocked with 5% (w/v) dried milk in tris-buffered saline with 0.1% Tween 20 (TBST) for 1 h. Membranes were incubated with primary Rabbit polyclonal recombinant Anti-RRAD antibody (Thermos Fisher, PA5-36460) 1:1000 in 3% BSA) overnight at 4°C, followed by three times wash with TBST. Membranes were then incubated with secondary antibodies (Goat anti-Rabbit IgG (H+L) Cross-Adsorbed Secondary Antibody), HRP (Thermo fisher # G-21234) 1:2500 in 5% milk) for 2 h at RT. The membrane was washed in TBST 3X. The expression was normalized to mouse GAPDH expression using Anti-GAPDH antibody [6C5] (Abcam # ab8245). Membrane proteins were detected and visualized using the ChemiDoc imaging system and analyzed using ImageJ software.

### Cellular Calcineurin Phosphatase Activity Assay

NMCM P7 were plated in 96 well plates and transduced with LacZ or RRAD adenovirus for 72 h. Cellular calcineurin phosphatase activity was then assessed following the manufacture protocol (Abcam # ab139464). First, a serial dilution of phosphate standard was prepared to produce a standard curve. The cells were washed with dH2O to remove any traces of the media. The cells in 96 well plate were divided into 4 groups: background group, Okadiac acid treated group, EGTA treated group, and positive control group following the manufacturing protocol. Then, the plate was incubated for 30 minutes at 30°C. The addition of malachite green reagent terminated the reaction and read on a microplate reader at OD 620nm_._

The standard curve was used to determine the amount of phosphate produced in each reaction. The calcineurin phosphatase activity was calculated as Calcineurin (PP2B) = Okadaic acid - (Okadaic acid + EGTA). The protein content in each well was also evaluated, and the calcineurin activity was calculated as ng phosphate per 1μg protein. The percentage of calcineurin activity in RRAD transduced NMCM P7 was calculated and compared to LacZ control NMCM P7 in each experiment.

### Heart-slicing and culturing

Donor hearts not utilized for cardiac transplantation were obtained through the United Network for Organ Sharing (UNOS). Slicing and culturing of 300 μm thick heart tissue slices were established in our lab and performed as previously described ^22,62,63^. Tissue slices were maintained in a refined oxygenated growth medium (Medium 199, 1x ITS supplement, 10% FBS, 5 ng/mL VEGF, 10 ng/mL FGF-basic, and 2x Antibiotic-Antimycotic (Invitrogen), which was changed 3 times/day. LacZ or RRAD *(*0.2 X10^7^ PFU*)* adenoviruses were added to the culture medium on the day of slicing. 48 h post-transduction, slices were transduced with CDK4, CCND (2F) adenoviruses (1 X10^7^ PFU each) and incubated for 48 h with media changes 3 times/day. Tissue slices were then processed for further analysis.

### Animal experiments

Animal studies were performed following the University of Louisville animal use guidelines, and the protocols were approved by the Institutional Animal Care and Use Committee (IACUC) and were accredited by the Association for Assessment and Accreditation of Laboratory Animal Care.

### MADM mice experiment

For lineage tracing, we used mosaic analysis with double markers (MADM). Transgenic mice were developed as described in ^20^. The mice were randomly selected to be injected with 20 μl containing LacZ (0.5X10^7^ PFU), RRAD 0.5X10^7^ PFU), 2F (5X10^7^ PFU each), and 2F +RRAD adenoviruses intramyocardially using a 30-gauge needle. The injections were made at the outer ventricular wall and the septum as two injections 10 μL each. 24 hours after injection, mice received Tamoxifen (40mg/kg I.P.) for three days (Sigma Aldrich T5648). Mice were euthanized 6 days after injection, and the hearts were harvested for pathology study. The mice sergeant was blinded to all administered viruses. MADM mice frozen hearts were sectioned longitudinally into 180–210 sections (3 sections per slide, collect one and throw away 2) 8μm thick. The frozen sections were fixed, permeabilized, and blocked as described in the Immunocytochemistry and immunohistochemistry section, then stained with Dapi 1μg/ml (Biotium Cat# 40043) to stain the nucleus. The coverslips were mounted with Vectashield antifading medium (vector labs Cat# H-1000) onto slides and visualized using the Keyence BZ9000 imaging system (10X magnification to the whole left ventricle). The percentage of single-colored cardiomyocytes from the total labeled cardiomyocytes was calculated using B.Z. Analyzer software. Individuals who analyzed the results were blinded to the treatment applied to each animal.

### Statistical Analyses

For all assays, power analyses were performed to choose the group sizes, which will provide >80% power to detect a 10% absolute change in the parameter with 5% Types I error rate. The Kolmogorov-Smirnov (K-S) test for normality was conducted. Then, differences between the two groups were examined for statistical significance with unpaired Student t-tests or Mann-Withney test. However, to compare data consisting of more than two groups, we performed one- or Two-way ANOVA tests followed by Dunnett’s post-test multiple comparisons to get the corrected p-value. A value of P<0.05 was regarded as significant, and error bars indicate standard deviation (SD).
